# Dietary Postbiotic *Lactobacillus plantarum* Improves Serum and Ruminal Antioxidant Activity and Upregulates Hepatic Antioxidant Enzymes and Ruminal Barrier Function in Post-Weaning Lambs

**DOI:** 10.3390/antiox9030250

**Published:** 2020-03-19

**Authors:** Wan Ibrahim Izuddin, Ali Merzza Humam, Teck Chwen Loh, Hooi Ling Foo, Anjas Asmara Samsudin

**Affiliations:** 1 Department of Animal Science, Faculty of Agriculture, Universiti Putra Malaysia, 43400 UPM Serdang, Selangor, Malaysiaanjas@upm.edu.my (A.A.S.); 2 Department of Animal Production, Faculty of Agricultural Engineering Sciences, University of Baghdad, Baghdad 10071, Iraq; 3 Institute of Tropical Agriculture and Food Security, Universiti Putra Malaysia, 43400 UPM Serdang, Selangor, Malaysia; 4 Department of Bioprocess Technology, Faculty of Biotechnology and Biomolecular Sciences, Universiti Putra Malaysia, 43400 UPM Serdang, Selangor, Malaysia; 5 Institute of Bioscience, Universiti Putra Malaysia, 43400 UPM Serdang, Selangor, Malaysia

**Keywords:** post-weaning lambs, postbiotics, antioxidant activity, lipid peroxidation, gene expression, hepatic antioxidant enzyme, ruminal barrier function

## Abstract

Postbiotics from *Lactobacillus plantarum* have been reported to improve growth performance, nutrient utilization, immune status and gut health in livestock. However, there is scarce information on the antioxidant activity of postbiotics and its modulation of antioxidant activity and rumen barrier function in animals. We investigated the antioxidant activity of postbiotics from *L. plantarum* RG14, RG11 and TL1 and dietary effects in post-weaning lambs on serum and ruminal antioxidant activity, hepatic antioxidant enzymes and ruminal barrier function. Postbiotic RG14 showed the highest antioxidant activity in both 2,2-diphenyl-1-picryl-hydrazyl (DPPH) and 2,2′-azino-bis (3-ethylbenzothiazoline-6-sulfonic acid) (ABTS) assay and was chosen to be evaluated in animal trials. Twelve post-weaning Dorper lambs were allotted to the control group and postbiotic group (0.9% (*v/w*) postbiotic RG14). The improvement in antioxidant activity of the postbiotic group was observed by greater glutathione peroxidase (GPX) in serum and ruminal fluid and lower serum TBARS. The findings were strengthened by the upregulation of hepatic *GPX1*, *GPX4* and copper, zinc superoxide dismutase (*Cu/Zn SOD*) in the postbiotic group. Lambs received postbiotics had higher regulation of rumen barrier function through upregulation of tight junction protein (*TJP*), occludin (*OCLD*), claudin-1 (*CLDN1*) and *CLDN4*. The current study demonstrated that dietary postbiotics enhanced the serum and ruminal fluid antioxidant activity, reduced the serum lipid peroxidation and upregulated hepatic antioxidant enzymes and ruminal barrier function.

## 1. Introduction

The exploration in finding the in-feed additives as alternatives to the use of antibiotics in livestock as a growth promoter has created huge attention due to the emergence of antimicrobial resistance. Recently, postbiotics, which are the secondary metabolites of probiotic bacteria, have been reported as great potential to substitute the use of antibiotics in animal production. The potential of postbiotics is ascribed to the existence of organic acids and antimicrobial compounds like bacteriocins, which possess inhibitory ability against important pathogenic bacteria including *Escherichia coli*, *Listeria monocytogenes*, *Pediococcus acidilactici, Salmonella typhimurium* and Vancomycin-resistant Enterococci (VRE) [1,2]. The dietary supplementation of postbiotics of monogastric like broilers [3,4,5], layer hens [6] and piglets [7,8] were shown to have better performance and were comparable or performed better in comparison to antibiotics. In broilers exposed to heat stress, postbiotics inclusion was shown to alleviate the heat stress effects by the greater growth performance, immune status and gut health [9]. Postbiotics use in ruminants was shown to improve the in vitro rumen fermentation and fiber degrading microbial population [10] and enhance growth performance, nutrient utilization, immune status and gut health in post-weaning lambs [11,12].

Peroxidation results from the crucial process of oxidative metabolism of cells, which in turn produces the reactive oxygens species and free radicals that lead to oxidative damage [13]. In normal conditions, the cells are protected by antioxidants and intracellular enzymes such as superoxide dismutase (SOD), glutathione peroxidase (GPX) and catalase (CAT), which eliminates peroxides and superoxides to prevent the formation of more reactive compounds by the reaction with metal catalysts [14]. Currently, in order to counter the oxidative damage, attention is given to postbiotics as probiotic-based additives with respect to the capacity to exhibit antioxidant potential with a wide variety of antioxidant enzyme activities. It has been proven by several studies that the probiotics from lactic-acid-producing bacteria exhibit high antioxidant activity [15,16,17]. High antioxidative capacity of the supplemented probiotics may modulate the antioxidant activity of the host. In mice models, probiotic *Bacillus licheniformis* and *B. velezensis* increased the serum total antioxidant, SOD and GPX and reduced the serum malondialdehyde (MDA) [18]. A higher level of serum total reduced glutathione (GSH) enzymes and serum total antioxidant was reported in growing Barki lambs supplemented with *Bacillus* spp. in drinking water [19]. Supplementation of lambs with probiotic *B. licheniformis* enhanced antioxidant capacity by elevating the SOD and GPX enzymes, as well as promoted immune function, which beneficially facilitates ruminal fermentation and microbial diversity [20]. This was also proven in our previous studies that postbiotics inclusion in the post-weaning Dorper lambs enhanced immune status and gut health and led to better nutrient utilization and growth performance [11,12].

The rumen epithelial barrier function through measurement of mRNA expression and protein concentration such as tight junction protein (TJP), claudin (CLDN) and occludin (OCLD) was studied in ruminants, but a majority of the studies do not focus on feed additive supplementation [21,22,23,24,25]. In modern ruminant production, grains inclusion in the diet increases fermentable carbohydrates and leads to excess accumulation of lactate and volatile fatty acids, which decrease rumen pH, elevate toxin concentration [26,27] and depress barrier function of gastrointestinal, leading to reduced nutrient absorption and production performance [21,28]. The stratified squamous epithelium of rumen is the major absorption site of crucial nutrients and highly selective to impede the concurrent entry of microorganisms and toxins from the ruminal lumen into the bloodstream, in which the absorption is transcellular and paracellular pathway is rigidly sealed [29]. The enclosed tight junction network that transfers tightness comprises OCLD, CLDN1, CLDN4 and tight junction-associated proteins [30].

Limited studies have been conducted on the use of *Lactobacillus plantarum* and its beneficial effects of antioxidant capabilities and barrier function in the rumen of ruminant animals. Considering the scarce information on this scope, the direction of the current study was to evaluate the potential of postbiotics produced from *L. plantarum* to deliver benefits of antioxidative properties as well as possible enhancement of ruminal barrier function. The *L. plantarum* is known to contain high antioxidative capacity [15,31,32]. The *L. plantarum* was shown to exhibit higher antioxidant activity in vitro among the *Lactobacillus* group isolated from fermented food and alleviate the negative effects of oxidized oil-induced hepatic injury in mice models [33]. We hypothesized that dietary postbiotics from *L. plantarum* modulates antioxidant activity and improves the regulation of barrier function in the lambs. Thus, this research aimed to study the effects of dietary postbiotic inclusion in post-weaning lambs on the serum and ruminal antioxidant activity and expression of genes related to hepatic antioxidant enzymes and ruminal barrier function. 

## 2. Materials and Methods

### 2.1. Microorganism Maintenance and Postbiotic Production

*L. plantarum* RG14, RG11 and TL1 were sourced at Industrial Biotechnology Laboratory, Department of Bioprocess Technology Faculty Biotechnology and Biomolecular Sciences, Universiti Putra Malaysia. The maintenance and reviving of bacterial cultures were detailed by Moghadam, et al. [34] and Foo, et al. [35] as the stock culture was prepared in 15% glycerol and kept at −80 °C for long-term storage. Bacterial inoculum was prepared by washing the active cultures with a sterile solution of 0.85% (*w/v*) NaCl (Merck, Darmstadt, Germany) and adjusted to 10^9^ CFU/mL. Then, the MRS media was inoculated by 10% (*v/w*) of 10^9^ CFU/mL active bacterial cells and incubated for 10 h at 30 °C, followed by the collection of supernatants by centrifugation (Benchtop Microfuge 20R, Beckman Coulter, Miami, FL, Germany) at 10,000× *g* at 4 °C for 15 min. The cell-free supernatant (CFS) was then collected by filtration through a 0.22 µm cellulose acetate membrane (Sartorius, Göttingen, Germany) [36]. The CFS was assayed immediately for antioxidant activity and the remaining kept at −20 °C until application in feed for feeding trial.

### 2.2. Total Antioxidant Activity of Postbiotics

Freshly collected postbiotics produced from *L. plantarum* RG14, RG11 and TL1 were quantified for total antioxidant activity of 2,2-diphenyl-1-picryl-hydrazyl (DPPH) and 2,2′-azino-bis (3-ethylbenzothiazoline-6-sulfonic acid) (ABTS) assay according to Chan, et al. [37].

#### 2.2.1. DPPH Radical Scavenging Assay 

Postbiotics were evaluated for DPPH free radical scavenging activity as described by Chan, et al. [37] with a slight adjustment based on the optimized ratio of sample to DPPH reagent. Briefly, 10 µL of the sample was reacted with 390 µL of 0.2 mM methanolic DPPH. The mixture was incubated for 60 min in dark and then centrifuged at 7500 rpm for 10 min at 15 °C. Two hundred microliters of supernatant were aliquoted into a microplate and the absorbance was measured at 517 nm with a Multiskan™ GO microplate photometer (Thermo Scientific™, Vantaa, Finland) against blank. The assay was conducted in triplicates. Ascorbic acid was used as a reference antioxidant. The scavenging activity of DPPH was calculated as below:

DPPH radical scavenging activity (%) = [(Ac − As)/Ac × 100]

where Ac is the absorbance of the control and As is the absorbance of the sample.

#### 2.2.2. ABTS^+^ Radical Scavenging Assay

The ABTS^+^ radical scavenging assay was conducted on postbiotics following the method described by Chan, et al. [37] with slight adjustment based on an optimized ratio of sample to ABTS reagent. The ABTS^+^ reagent was prepared by reacting 50 mL of 7 mM ABTS stock solution with 50 mL of 2.45 mM potassium persulfate and kept in dark at room temperature for 2 h. The ABTS+ solution was diluted to reach absorbance of 0.70 ± 0.02 at 732 nm. The diluted sample was mixed with 190 µL of adjusted ABTS^+^ solution in a microplate and then kept in dark for 10 min at room temperature. The absorbance was measured at 734 nm with Multiskan™ GO microplate photometer (Thermo Scientific™, Vantaa, Finland) against blank. Ascorbic acid was used as a reference antioxidant. The scavenging activity of ABTS^+^ was calculated as below:

ABTS^+^ radical scavenging activity (%) = [(Ac − As)/Ac × 100]

where Ac is the absorbance of the control and As is the absorbance of the sample.

### 2.3. Feeding Trial and Sample Collection

The protocol of the feeding trial was approved by Universiti Putra Malaysia Research Committee that the use and care of animals for scientific purposes are humane and ethical according to Institutional Animal Care and Use Committee (IACUC) UPM (No. 9525500: Effects of inclusion of postbiotics from *Lactobacillus plantarum* in post-weaning lambs). The feeding trial was conducted at the Ruminant Unit, Department of Animal Science Research Farm, Universiti Putra Malaysia. Detailed information on management and feeding of post-weaning lambs have been reported in our companion papers [11,12]. Briefly, twelve male lambs (17.3 ± 0.58 kg, 16 weeks old) were randomly divided to the control group (no postbiotic inclusion) and postbiotic group (0.9% (*v/w*) postbiotic RG14 inclusion). The postbiotic RG14 was chosen based on the highest antioxidant activity by ABTS and DPPH assay and a 0.9% level of postbiotic RG14 inclusion was selected according to in vitro findings reported by Izuddin, et al. [10]. The lambs were individually penned and fed the treatment diets for 60 days including the adaptation period for 14 days. The ingredient compositions and nutrient constituents of the treatments are shown in Table 1. Drinking water was available at all times at each pen.

Five milliliters of blood were drawn into Serum BD Vacutainer^®^ (BD, Franklin Lakes, NJ, USA) blood collection tubes by jugular venepuncture at day 58 of the feeding trial period. Blood samples were allowed to coagulate and then centrifuged at 3000× *g* for 15 min and stored at −80 °C until further analysis. All lambs were dispatched on the last day of feeding trial to the abattoir at the Department of Animal Science, Universiti Putra Malaysia and fasted for 12 h at the lairage with the access of freshwater. The lambs were sacrificed by severing the carotid artery and jugular vein following the Halal slaughtering procedure (MS1500: 2009) as described by the Malaysian Standard [38]. Immediately after evisceration, the left lobe of the liver and caudal ventral of rumen tissues were collected and kept in liquid nitrogen before transferring to −80 °C freezer upon sample analysis. Rumen fluid was collected and strained immediately with cheesecloth and kept directly into ice and transferred into −80 °C freezer until analysis.

### 2.4. Serum and Ruminal Fluid Antioxidant Enzymes Activity

Thiobarbituric acid reactive substances (TBARS) assay measured as MDA as a product of lipid peroxidation and antioxidant enzymes of GPX and SOD were measured in the serum and ruminal fluid of the lambs using assay kits (Elabscience Biotechnology, Houston, TX, USA) by following manufacturer’s instruction.

### 2.5. Gene Expression 

Total RNA extraction of rumen and liver tissues was performed using RNeasy^®^ Mini Kit (Qiagen, Hilden, Germany), according to the protocol provided by the manufacturer. The concentration and purity of RNA were determined using Nanodrop 2000 spectrophotometer (Thermo Scientific, Wilmington, DE, USA). The purified RNA at a concentration of 1000 ng/µL was transformed into complementary DNA (cDNA) using the Quantitect^®^ Reverse Transcription kit (Qiagen, Hilden, Germany) according to the protocol provided by the manufacturer. The primers of the target genes were designed using Primer 3 Plus and synthesized (BioNeer Corporation, Oakland, CA, USA) based on *Ovis aries* sequences in the GenBank. The detailed information of the primers is presented in Table 2. Quantitative PCR was conducted using the Bio-Rad CFX96 Real-time PCR system (Bio-Rad Laboratories, Hercules, CA, USA). Table 3 shows the target and reference genes’ primer sequence and product size. Temperature gradient protocol was conducted to identify the ideal annealing temperature of reference and target genes using a similar machine. The qPCR cycling condition involved heat activation for 10 min at 95 °C, followed by 40 cycles of denaturation for 15 s at 95 °C, annealing for 30 s at 57 °C for *GAPDH*, *CLDN4*, 60 °C for *TJP*, *OCLD*, *CLDN1* and 61 °C for *Cu/Zn SOD*, *GPX1* and *GPX4* and, finally, extension for 30 s at 72 °C. The specificity of PCR amplification was conducted at the completion 40-amplification cycle. The relative expression of target genes was calculated according to the method described by Livak and Schmittgen [39]. The *GAPDH* gene was used as an internal standard (housekeeping gene) to standardize the expression of target genes as reported by Saeed, et al. [40] as it provided stable expression for sheep liver and gut tissues. The efficiency of amplification of genes was calculated by plotting a standard curve of 5-fold serial dilution of cDNA.

### 2.6. Statistical Analysis

Completely randomized design (CRD) was applied for the experiments. The independent *t*-test was used to identify the differences between treatments using the PROC TTEST procedure of Statistical Analysis System software version 9.2 (SAS Institute, Cary, NC, USA) and the differences were considered significant at *p* < 0.05.

## 3. Results

### 3.1. Antioxidant Activity of Postbiotics

Among the postbiotic *L. plantarum*, RG14 strain showed highest antioxidant activity ABTS radical scavenging assay as compared with RG11 and TL1 (Table 3). No difference between different postbiotics in DPPH assay, however, RG14 showed numerically higher antioxidant activity as compared to RG11 and TL1. Because of the higher in total antioxidant activity, only postbiotic RG14 was chosen to be further evaluated in the feeding trial of post-weaning lambs.

### 3.2. Antioxidant Enzyme Activity

**Figure 1:**
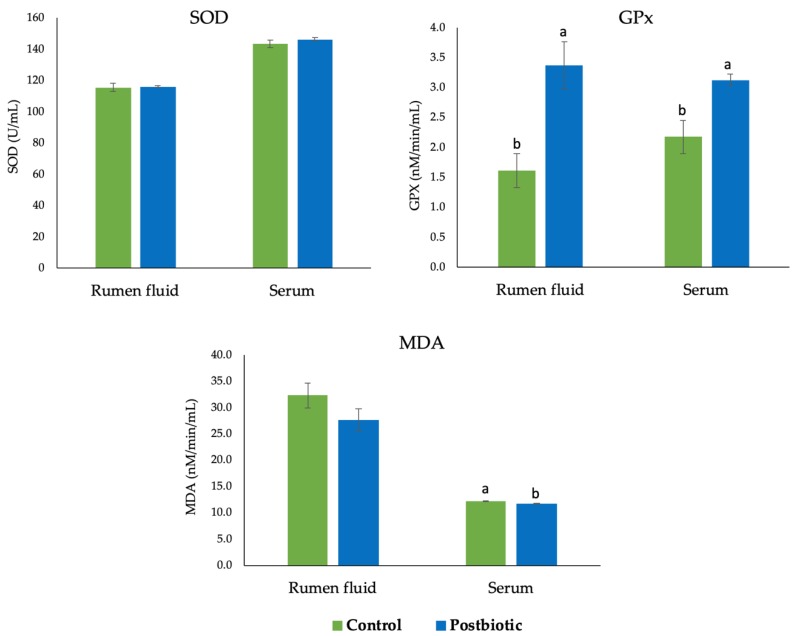
Serum antioxidant activity in post-weaning lambs supplemented with postbiotics from *L. plantarum* RG14. SOD: Superoxide dismutase, GPX: Glutathione peroxidase, MDA: Malondialdehyde. ^a,b^ Bar with different letters between treatment groups are significantly different (*p* < 0.05).

Dietary postbiotic *L. plantarum* RG14 did not affect (*p* > 0.05) serum superoxide dismutase (SOD) in both rumen fluid and serum of lambs (Figure 1). However, serum and ruminal glutathione peroxidase (GPX) enzymes were higher in the postbiotic group. No difference was recorded in the malondialdehyde (MDA) concentration in rumen fluid but lower MDA was observed in serum of the postbiotic group.

### 3.3. Gene expression of Hepatic Antioxidant Enzymes 

**Figure 2:**
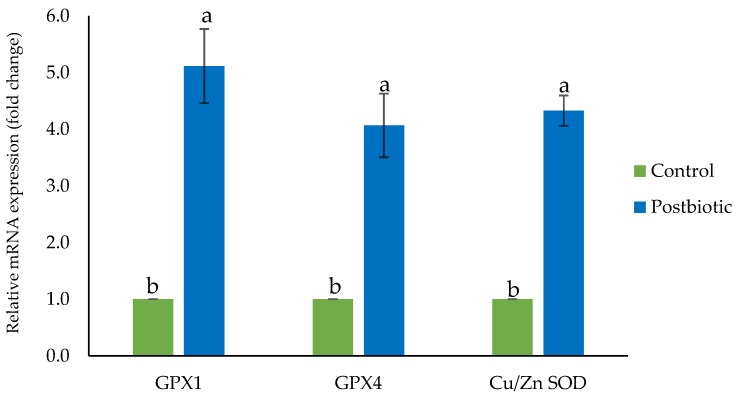
Gene expression of hepatic antioxidant enzymes of post-weaning lambs supplemented with and without postbiotic *L. plantarum* RG14. *GPX1*: Glutathione peroxidase 1, *GPX4*: Glutathione peroxidase 4, *Cu/Zn SOD*: Cu, Zn Superoxide dismutase. ^a,b^ Bars with different letters between treatment groups are significantly different (*p* < 0.05).

Supplementation of postbiotic *L. plantarum* RG14 in the diet of post-weaning lambs gave great impacts on the regulation of hepatic antioxidant enzyme genes. Postbiotic inclusion upregulated (*p* < 0.05) the expression of *GPX1*, *GPX4* and *Cu/Zn SOD* genes 5.1-, 4.0- and 4.3-folds, respectively (Figure 2).

### 3.4. Gene Expression of Ruminal Barrier Function

**Figure 3:**
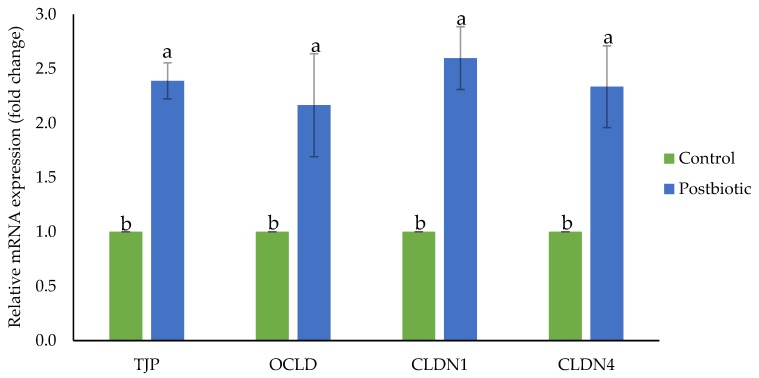
Gene expression of the ruminal barrier function of post-weaning lambs supplemented with and without postbiotic *L. plantarum* RG14. TJP: Tight junction protein, OCLD: Occludin, CLDN1: Claudin 1, CLDN4: Claudin 4. ^a,b^ Bars with different letters between treatment groups are significantly different (*p* < 0.05).

The inclusion of postbiotics in post-weaning lambs positively affected the expression of tight junction proteins that regulate ruminal barrier function. Upregulation of *TJP*, *OCLD*, *CLDN1* and *CLDN4* was observed in the rumen epithelium of lambs supplemented with postbiotics (Figure 3).

## 4. Discussion

The production of reactive oxygen species (ROS) and oxygen-centered free radicals as by-products results from biochemical and physiological processes [33,41]. High levels of ROS due to either an increased production of oxidant species or a decreased efficacy of the antioxidant system can lead to oxidative stress, an emerging health risk factor involved in many diseases, both in humans and in animals [42]. The excessive creation and accumulation of ROS such as hydrogen peroxide, hydroxyl radicals and superoxide anion radicals may destruct DNA, lipids and proteins, leading to the destruction of tissue and later cause organ failure [43]. It is well-versed that the enzymatic defense by antioxidant enzymes is crucial to protect the bacteria from oxidative stress. The presence of antioxidant activity of lactic acid bacteria can be owing to the production of exopolysaccharides, lipoteichoic acid and cell-surface proteins [31,33,44,45]. The *Lactobacillus plantarum* strains were shown to exhibit high antioxidant activity by showing high resistance to hydrogen peroxide and high scavenging activity against hydroxyl, superoxide and DDPH free radicals [31,46]. Postbiotics are rich in organic acids mainly lactic acid and acetic acid [1,2,47]; the acids are great electron donors due to the existence of hydroxyl groups that increase the free radical scavenging activity [48]. The cell-free extracts such as postbiotics contain intracellular enzymes produced by *L. plantarum* and the bacterial cell destruction prior to the collection of cell-free supernatant released the enzymes. Kullisaar, et al. [16] and Lin and Yen [49] demonstrated that several cell-free extracts of lactic acid bacteria strains showed in vitro antioxidant activity. In defending against oxidative stress, the enzymatic defense system of lactic acid bacteria involves the production of intracellular antioxidant enzymes like superoxide dismutase (SOD), NADH-oxidase and NADH-peroxide, in addition to heterologous non-haem catalase [31]. The DPPH and ABTS free radical scavenging assay confirmed the antioxidative properties of postbiotics produced by *L. plantarum*. In respect to this, it potentiates the supplementation of postbiotics based as feed additives in animals. 

Therefore, the ability of postbiotics harvested from *L. plantarum* to alleviate oxidative stress in post-weaning lambs was evaluated in this study. In the current study, we found a higher GPX enzyme concentration in both serum and rumen fluid in post-weaning lambs that received postbiotics in their diet. A similar pattern of findings was shown by the hepatic antioxidant enzyme in the upregulation of *GPX1*, *GPX4* and *Cu/Zn SOD* enzyme genes in the liver of the postbiotic group. The SOD and GSH-Px are intracellular antioxidant enzymes that are involved in the enzymatic antioxidant system against oxidative stress [50] and are essential buffers in the interference and deterioration of superoxide anion and hydrogen peroxide in cells [31]. MDA is produced as the final product in lipid peroxidation and the level often associated as an indicator of aging and oxidative damage. It may react with biological molecules and exert genotoxic and cytotoxic effects in organisms [31]. We found lower serum MDA levels in post-weaning lambs that consumed in-feed postbiotics, reflecting the protective effects of dietary postbiotics in reducing the peroxidation of lipid. The postbiotics contain the secondary metabolites of the *L. plantarum* including the organic acids, bacteriocin proteins and enzymatic and non-enzymatic antioxidant compounds, which convey similar actions and effects of the bacteria itself. The antioxidative modulation of probiotic bacteria involves the chelation of metal ion, production of own antioxidases, generation of antioxidant metabolites, upregulation of antioxidase action and enhancement of antioxidant metabolites of the host, coordinate the signaling pathway, reduction of the enzyme activities of reactive oxygen species and regulation of microbiota of gastrointestinal tract [48]. Identical findings were reported by Jia, et al. [20] of a greater concentration of serum SOD and GPX enzymes in lambs supplemented with *Bacillus licheniformis*. The enhanced antioxidant capacity was in concert with the promoted immune function that beneficially facilitates ruminal fermentation and microbial diversity. A similar trend of findings with supplementation of *L. plantarum* was also published in other species. Inclusion of *L. plantarum* in the diet of post-weaning piglets was demonstrated to alleviate oxidative stress by an increase in the serum concentration of SOD, GPX and CAT and reduce serum MDA [46]. In growing-finishing pigs, *L. fermentum* supplementation enhanced serum SOD and GPX and increased hepatic CAT, muscle SOD and Cu and Zn SOD [51]. The serum and liver concentration of GPX, SOD, glutathione (GSH) and glutathione reductase (GR) were increased and the MDA level in the serum and liver was decreased in broilers fed with *Bacillus subtilis* in the diet [52]. In mice, administration of *L. plantarum* escalated the serum SOD activity, increased hepatic GPX activity and total antioxidant capacity in the liver, while lower the level of hepatic MDA [31]. 

Sustaining the integrity of the gastrointestinal barrier function is central in promoting the health of the gut ecosystem as it prevents the invasion of allergens, toxins and pathogens and preserves homeostasis between commensal microbes and the immune system. In vitro and in vivo studies have provided promising evidence of *Lactobacillus* species being beneficial in sustaining the integrity of the tight junction and barrier function and having therapeutic benefits in the medication of gastrointestinal related diseases [53]. Postbiotics contain secondary metabolites of the *L. plantarum* and transmit the properties and abilities of probiotic bacteria. The interaction of metabolites and bioactive molecules produced by probiotics strengthens the integrity of the tight junction and counters the disruption caused by induced destructive factors [53,54]. To the best of our knowledge, our study is the first to evaluate the effects of dietary postbiotic on the regulation of rumen tight junction proteins in post-weaning ruminants. Upregulation of tight junction protein genes was observed in the rumen of post-weaning lambs receiving dietary postbiotic. Postbiotic supplementation in the diet of post-weaning lambs was reported to improve the intestinal barrier function simultaneously with improvement in intestinal morphology, microbial population and mucosal immunity [11]. Studies on *L. plantarum* as feed additives in another species showed improvement of gut barrier function accompanied by a better gut environment. Post-weaning piglets received *L. plantarum* in the diet had improvement in the regulation of tight junction proteins (claudin-1, occludin and zona-occluden-1 (*ZO-1*)) concurrently with the strengthening of epithelium and modulating gut microbiota [55]. In suckling piglets, *L. plantarum* administration in the diet upregulated the jejunal *OCLD*, ileal *ZO-1* and jejunal and the ileum of porcine B-defensin with an improvement of intestinal morphology and increment of relative abundance of phyla *Firmicutes* and *Actinobacteria* and genus *Lactobacillus* [56]. Orally supplemented mice with *L. plantarum*-fermented milk modulated the intestinal barrier function and ameliorated the enteric infection by *Salmonella* through the evaluation of intestinal permeability and histopathology [57]. In mammalian cell culture, (Caco-2) treated with *L. plantarum* altered in expression of the network of genes related to the tight junction and higher intensity of immuno-stained occludin, *ZO-1*, *ZO-2* and cingulin was visualized by fluorescent microscopy [58]. Most of the studies have found the positive effects of dietary *L. plantarum* supplementation on gut barrier function consistently accompanied by improvement in gut microbiota, mucosal immunity and morphology.

## 5. Conclusions

In conclusion, postbiotics particularly RG14 exhibited higher antioxidant capacity in which dietary to the post-weaning lambs is beneficial to deliver antioxidant capacity and reduce oxidative stress. Postbiotic *L. plantarum* RG14 supplementation in post-weaning lambs showed higher antioxidant enzymes in serum and ruminal fluid and lower lipid peroxidation in serum. These findings were further evidenced by the upregulation of hepatic genes of antioxidant enzymes and lead to the improvement of the regulation of tight junction proteins in rumen epithelium. Dietary postbiotics as feed additives has a bright potential to be used to reduce oxidative stress in post-weaning lambs and provide better protection to the gut barrier function.

## Figures and Tables

**Table 1:** Feed and nutrient composition.

	Control	Postbiotic
Feed composition (%)		
Grass	30.00	30.00
Corn	40.00	40.00
Soybean meal	23.80	23.80
Wheat pollard	3.40	3.40
Palm oil	0.90	0.90
Calcium carbonate	1.70	1.70
Sodium chloride	0.40	0.40
Mineral premix ^1^	0.90	0.90
Vitamin premix ^2^	0.90	0.90
Postbiotic RG14	-	0.90
Nutrient composition (% DM)		
ME (MJ/kg)	8.09	8.09
Crude protein	16.90	16.90
Crude fat	2.70	2.67
NDF	59.60	59.60
ADF	16.90	16.60

^1^ The mineral premix supplies approximately 22.5 mg Co, 1.35 g Cu, 7.2 g Fe, 90 mg I, 360 mg K, 9 g Mn, 18 mg, Se, 7.2 g Zn per kg of feed. ^2^ The vitamin mix supplies approximately 0.45 MIU vitamin A, 0.09 g vitamin B1, 0.27 g vitamin B2, 0.18 g vitamin B6, 0.09 mg vitamin B12, 0.09 MIU vitamin D3, 0.67 g vitamin E, 0.18 g vitamin K3, 2.12 mg biotin per kg of feed. ME: Metabolizable energy, NDF: Neutral detergent fiber, ADF: Acid detergent fiber. The formulation of diets was conducted using FeedLive Software (Live Informatics, Nonthaburi, Thailand).

**Table 2:** Primer information of reference and target genes.

Gene	Primer Sequence (5′-3′)	Product Size (bp)	NCBI Accession Number
*Cu/Zn SOD*	F-GAC TTG GGC AGA GGT GGA AA R-CAG GGA ATG TTT ACG GGG CA	100	NM_000454.4
*GPX1*	F-CCT GGT CGT ACT CGG CTT C R-CCT TCT CGC CAT TCA CCT C	154	NM_000581.3
*GPX4*	F-GGG AGT AAT GCG GAG ATC AA R-CAT ACC GCT TCA CCA CAC AG	210	NM_001039847.2
*TJP1*	F-CGACCAGATCCTCAGGGTAA R-AATCACCCACATCGGATTCT	161	XM_015101949.1
*OCLD*	F-GTTCGACCAATGCTCTCTCAG R-CAGCTCCCATTAAGGTTCCA	196	XM_015101256.1
*CLDN1*	F-CACCCTTGGCATGAAGTGTA R-AGCCAATGAAGAGAGCCTGA	212	NM_001185016.1
*CLDN4*	F-AAGGTGTACGACTCGCTGCT R-GACGTTGTTAGCCGTCCAG	237	NM_001185017.1
*GAPDH*	F-ACCACTTTGGCATCGTGGAG R-GGGCCATCCACAGTCTTCTG	76	NM_001190390.1

F: Forward, R: Reverse, *Cu/Zn SOD*: Cu, Zn Superoxide dismutase, *GPX1*: Glutathione peroxidase 1, *GPX4:* Glutathione peroxidase 4, *TJP1*: Tight junction protein 1, *OCLD*: Occludin, *CLDN1*: Claudin 1: *CLDN4*: Claudin 4, *GADPH*: glyceraldehyde-3-phosphate dehydrogenase.

**Table 3:** 2,2-diphenyl-1-picryl-hydrazyl (DPPH) and 2,2′-azino-bis (3-ethylbenzothiazoline-6-sulfonic acid) (ABTS) radical scavenging activity of postbiotics from *L. plantarum* TL1, RG11 and RG14.

Postbiotics	TL1	RG11	RG14	SEM	*p*-Value
DPPH (µg AAEAC/mL)	67.04	72.295	74.284	1.8102	0.266
ABTS (µg AAEAC/mL)	151.822 ^b^	150.617 ^b^	202.831 ^a^	8.7876	<0.0001

AAEAC: Ascorbic acid equivalents antioxidant capacity. ^a,b^ Means with different superscript in row are significantly different (*p* < 0.05).
